# Editorial: Transforming veterinary medicine: digital tools and AI as path to sustainable animal care

**DOI:** 10.3389/fvets.2026.1930104

**Published:** 2026-08-04

**Authors:** Andra-Sabina Neculai-Valeanu, Vasile M. Maciuc

**Affiliations:** 1 Romanian Academy, Iaşi Branch, Department of Knowledge Ecosystems, Iaşi, Romania; 2 Faculty of Food and Animal Sciences, Department of Animal Resources and Technologies, Iaşi University of Life Sciences “Ion Ionescu de la Brad”, Iaşi, Romania

**Keywords:** animal health, animal welfare, artificial intelligence, companion animals, digital health monitoring, digital tools, precision livestock farming, wildlife management

Veterinary medicine is undergoing a transformation driven by the increasing availability of advanced technologies. In particular, the combination of artificial intelligence, IoT devices, and sensor-rich environments is changing the practice of veterinary medicine from episodic to continuous monitoring relevant to decision-making. This Research Topic brings together 15 contributions dealing with precision livestock farming, diagnostics and care of companion animals, wildlife and population management, veterinary education, professional adoption and ethics, and environmental sustainability Together, these articles depict a field that is progressing from the proof-of-concept phase toward the development of impactful and responsible digital solutions, while consistently underscoring the fact that the impact of these solutions depends not only on their technical performance but also on their scalability, cost-effectiveness, and ethical deployment and adoption by the veterinary and animal care communities.

One of the significant themes in this Research Topic of papers is the non-invasive data-driven phenotyping at the farm level. Lameness is considered one of the most difficult challenges in dairy cattle welfare and productivity. Duan et al. proposed accurate keypoint localization with multi-feature fusion for effective lameness classification using overhead RGB-D surveillance. Their research integrated precise keypoint localization with multi-feature fusion to support robust lameness classification from overhead RGB-D surveillance, enabling unobtrusive, continuous monitoring without disrupting animal behavior. Complementing vision-based approaches, audio analytics offer a low-cost route to welfare assessment. Jobarteh et al., combined classical acoustic features with transformer-based representations to advance behavior classification and real-time stress flagging in dairy systems. In pigs, Hao et al. demonstrated how architectures fusing spatial and temporal representations improve behavior recognition, an upstream capability for illness detection, welfare surveillance, and environment optimization. Extending non-invasive sensing to poultry, Dhaliwal et al. demonstrated, in a pilot study of early-life laying hens, that a stratified multimodal approach integrating thermal imaging, acoustics, optical-flow movement metrics, and environmental data can capture developmentally meaningful welfare signals from hatch onward, illustrating both the promise and the annotation-workload constraints of multimodal monitoring.

Parallel to these computational breakthroughs, a comprehensive review by Abdel-Wareth et al. mapped AI applications in rabbit husbandry from reproductive monitoring to precision farming while surfacing common adoption bottlenecks, such as data capture, ethics, affordability, and integration across diverse farm conditions, that apply broadly across species and sectors.

The Research Topic also advances diagnostic specificity in companion animal medicine. Heterogeneous clinical presentations and complex workups can significantly delay targeted treatment. Reichert et al. applied deep-learning neural networks to routine B-mode ultrasonography as a decision-support tool for identifying protein-losing enteropathy imaging features in dogs, exemplifying how imaging AI can supplement diagnostic pathways already embedded in practice. Vision-based methods are also maturing for musculoskeletal assessment: in a state-of-the-art review, Phelan et al. examined computer-vision approaches to canine gait analysis, highlighting the potential of single-camera pose reconstruction to extend objective assessment beyond specialized laboratories into domestic environments, while identifying the biomechanical-fidelity, standardization, and validation gaps that remain before clinical deployment.

Digital tools are equally relevant beyond the clinic and the farm, in wildlife health and population management, a core concern of zoological medicine. Reliable individual identification is a prerequisite for managing free-ranging populations, yet it is often invasive and logistically demanding. Pereira and Rutberg evaluated radio-frequency identification (RFID) as a less invasive alternative for identifying individual white-tailed deer (*Odocoileus virginianus*) enrolled in porcine zona pellucida (PZP) immunocontraceptive programs. Using passive integrated transponder tags in 52 wild deer together with fixed reader stations, they showed that tags were reliably detected at close range and remained readable years after implantation, pointing toward a future in which a single dart could deliver both an identification tag and a fertility-control agent, thus reducing handling stress and improving the feasibility of humane, technology-enabled wildlife population management.

Digital transformation also includes care pathways, communication, and public engagement aspects of practice, as seen in the case of veterinary telemedicine, which represents one of the most conspicuous changes in small-animal practice. A parallel mixed-method approach to a SWOT analysis, as carried out through a study on “*Veterinary telemedicine in small animal practice in Germany*” based on pet owners' survey results and experts' views, provided a comprehensive overview of the opportunities, constraints, and threats associated with this practice, including aspects such as “access, flexibility, and stress reduction” as opportunities, “no physical examination, technical barriers, legal situation, and data protection” as constraints, and “increase of telemedicine, more veterinarians offering telemedicine services“ as potential threats. These findings highlight the importance of governance and stakeholder alignment as prerequisites for sustainable adoption (Karsch et al.). From a One Health perspective, work carried out by de Castro et al. examining zoonoses knowledge among dog and cat owners in Rio de Janeiro, alongside patterns of internet and app use, showcased that mobile-first tools may strengthen health education when they complement, rather than displace, professional veterinary guidance.

As far as education and workforce are concerned, documentation remains a constraint, as seen in the study entitled “*Comparison of smartphone-native transcription, Whisper-based transcription, and large language model summarization of SOAP format educational text*” carried out by Yogo, who demonstrated the potential of AI in creating educational materials, while at the same time highlighting the potential of “semantic distortion” associated with AI-generated materials. Complementary, a mini-review by Li et al. highlights curriculum redesign through AI-enabled knowledge structures and blended learning loops, addressing an under-discussed dimension of digital transformation: training clinicians to reason effectively alongside data-driven tools.

Realizing this potential ultimately depends on trust, adoption, and ethical governance. Surveying 455 veterinary professionals in China, Li and Lai identified a striking “*adoption paradox*.” A majority already incorporate AI into their workflows, yet many active users report low familiarity with the technology, and concerns about reliability and accuracy remain the primary barrier, findings accompanied by strong support for regulatory oversight by veterinary authorities. These tensions make the ethical dimension explicit. In a focused review, Heinlein argued that AI should aid, not replace, veterinary decision-making, and identified the accuracy and reliability of AI interpretations, algorithmic bias, data privacy, and the balance of innovation with legal and professional responsibilities as the key areas requiring foundational standards, education, and continued research, particularly for clinically ambiguous cases.

Environmental sustainability is the final dimension addressed in this Research Topic. Veterinary teams are seeking means to reduce their clinical footprint. Studies such as the one carried out by Kern-Allely et al. synthesized best practices across energy efficiency, procurement, anesthetic gas management, waste reduction, and community engagement, providing a practical roadmap for both incremental improvement and longer-horizon change.

Taken together, these papers support an overarching theme: digital veterinary medicine is not about the development of models but rather the engineering of workflows. A tool that is effective in a controlled setting must be effective in a multi-site prospective study; it must be interoperable, auditable, and non-intrusive; and it must decrease, not increase, the workload of the veterinarian and the animal caretakers. The next phase of this field calls for convergence across sensing, analytics, service delivery, education, and environmental practice—with responsible AI as the common thread. The shared objective is clear: measurably improved animal welfare and care quality, achieved in ways that are ethically sound and operationally sustainable across the full spectrum of veterinary medicine.

